# Radiosynthesis, *In Vivo* Biological Evaluation, and Imaging of Brain Lesions with [^**123**^I]-CLINME, a New SPECT Tracer for the Translocator Protein

**DOI:** 10.1155/2015/729698

**Published:** 2015-06-25

**Authors:** F. Mattner, M. Quinlivan, I. Greguric, T. Pham, X. Liu, T. Jackson, P. Berghofer, C. J. R. Fookes, B. Dikic, M.-C. Gregoire, F. Dolle, A. Katsifis

**Affiliations:** ^1^Life Sciences Division, Australian Nuclear Science and Technology Organisation, New Illawarra Road, Lucas Heights, NSW 2234, Australia; ^2^Department of Molecular Imaging, Royal Prince Alfred Hospital, Camperdown, NSW 2050, Australia; ^3^CEA, DSV/I2BM, Service Hospitalier Frédéric Joliot, 4 Place du Général Leclerc, 91401 Orsay, France

## Abstract

The high affinity translocator protein (TSPO) ligand 6-chloro-2-(4′-iodophenyl)-3-(*N,N*-methylethyl)imidazo[1,2-a]pyridine-3-acetamide (CLINME) was radiolabelled with iodine-123 and assessed for its sensitivity for the TSPO in rodents. Moreover neuroinflammatory changes on a unilateral excitotoxic lesion rat model were detected using SPECT imaging. [^123^I]-CLINME was prepared in 70–80% radiochemical yield. The uptake of [^123^I]-CLINME was evaluated in rats by biodistribution, competition, and metabolite studies. The unilateral excitotoxic lesion was performed by injection of *α*-amino-3-hydroxy-5-methylisoxazole-4-propionic acid unilaterally into the striatum. The striatum lesion was confirmed and correlated with TSPO expression in astrocytes and activated microglia by immunohistochemistry and autoradiography. *In vivo* studies with [^123^I]-CLINME indicated a biodistribution pattern consistent with TPSO distribution and the competition studies with PK11195 and Ro 5-4864 showed that [^123^I]-CLINME is selective for this site. The metabolite study showed that the extractable radioactivity was unchanged [^123^I]-CLINME in organs which expresses TSPO. SPECT/CT imaging on the unilateral excitotoxic lesion indicated that the mean ratio uptake in striatum (lesion : nonlesion) was 2.2. Moreover, TSPO changes observed by SPECT imaging were confirmed by immunofluorescence, immunochemistry, and autoradiography. These results indicated that [^123^I]-CLINME is a promising candidate for the quantification and visualization of TPSO expression in activated astroglia using SPECT.

## 1. Introduction

The 18 kDa translocator protein [1] (TSPO; previously known as the peripheral benzodiazepine receptor, PBR) has been extensively studied for its intricate biological functions. Although the physiological role of the TSPO is still unclear, its primary location in the outer membrane of the mitochondria and its role in the energy production, maintenance of membrane potential, cellular respiration, and regulation of steroidogenesis are well documented [2].

Most intriguing are the TSPO's relatively low concentrations in the CNS, particularly in microglia and astrocytes [3, 4] that upon insult become activated resulting in significant overexpression in these cell types [5–7]. Consequently, TSPO overexpression has been confirmed in Alzheimer's disease (AD), multiple sclerosis, stroke, Parkinson's disease, HIV encephalitis, and trauma [7–11]. The observation that TSPO are also found in cancer cells [12] also suggests a role in cell proliferation, malignancy, and modulation of apoptosis [13, 14]. As a consequence the TSPO has been targeted for both potential therapeutic applications and in imaging using Positron Emission Tomography (PET) and Single Photon Emission Computed Tomography (SPECT).

The isoquinoline carboxamide PK11195, radiolabelled with tritium [3], carbon-11 [15], iodine-123/125 [16–18], and fluorine-18 [19] have been extensively used as pharmacological probes for studying the function and expression of the TSPO. As a result, these compounds have been shown to localise in areas associated with microglia activation following a range of neurological insults [7] and a number of PET and SPECT clinical studies have been performed using [^11^C]-PK11195 [20–22] and [^123^I]-PK11195 [23] to detect microglia activation in humans.

However, these radiolabelled PK11195 analogues displayed a significant degree of nonspecific binding* in vivo* [24], a characteristic which can distort the imaging data. This has driven research towards the development of other classes of high affinity TSPO ligands [25–28] for use in PET and SPECT imaging.

**Figure 1:**
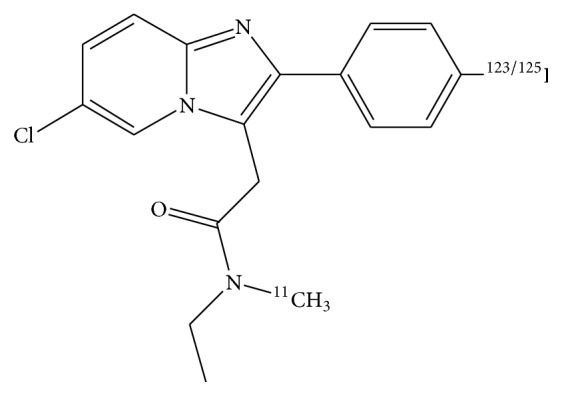
Structure of CLINME.

The acetamide structure of CLINME provides a unique opportunity to prepare structurally diverse compounds suitable for the incorporation of a variety of PET and SPECT isotopes. Consequently, *N*′,*N*′-methylethyl-6-chloro-(4′-iodophenyl)imidazo[1,2-a]pyridine-3-acetamide or CLINME (Figure 1) has been prepared for radiolabelling with both carbon-11 and iodine-123 for PET and SPECT studies, respectively. We have shown that [^11^C]-CLINME [29, 30] has demonstrated lower nonspecific binding than [^11^C]-PK11195 and was better able to depict microglia activation following unilateral excitotoxic lesions in rodent models of inflammation than [^11^C]-PK11195 [31]. As this molecule also incorporates iodine, it presents an excellent opportunity to also exploit additional studies when radiolabelled with iodine-125 for pharmacological studies as well as with iodine-123 and iodine-124 for use in SPECT and PET imaging, respectively. Here, we report the radiolabelling,* in vitro, in vivo* pharmacological evaluation of the iodine-123 labelled analogue [^123^I]-CLINME and its* in vivo* imaging in a model of the unilateral excitotoxic lesion using SPECT.

## 2. Materials and Methods

### 2.1. Synthesis and Characterisation of Chemical Precursors and Standards

NMR spectra were performed on a Bruker Avance DPX 400 operating at 400 MHz for ^1^H NMR spectra and 100 MHz for ^13^C. Chemical shifts are given in ppm (*δ*) from the internal standard tetramethylsilane in the specified deuterated solvents. Low resolution mass spectra were performed using either a VG Quattro triple quadrupole mass spectrometer (Fisons Biotech MS, Altrincham, UK) in electrospray mode or a Micromass ZMD Quadrupole Mass Spectrometer for Electron Impact.

Melting points were performed on a Gallenkamp melting point apparatus and are uncorrected. Elemental analysis was performed on a Carlo Erba 1106 Elemental Analyser by the Australian National University (Canberra, Australia) and results were within ±0.40% of the theoretical value.

All reagents and solvents if not specified were purchased from Sigma-Aldrich and were used without further purification. The structures of the synthesised compounds were confirmed by ^1^H NMR, ^13^C NMR, MS, and microanalysis (or HRMS).

**Figure 2:**
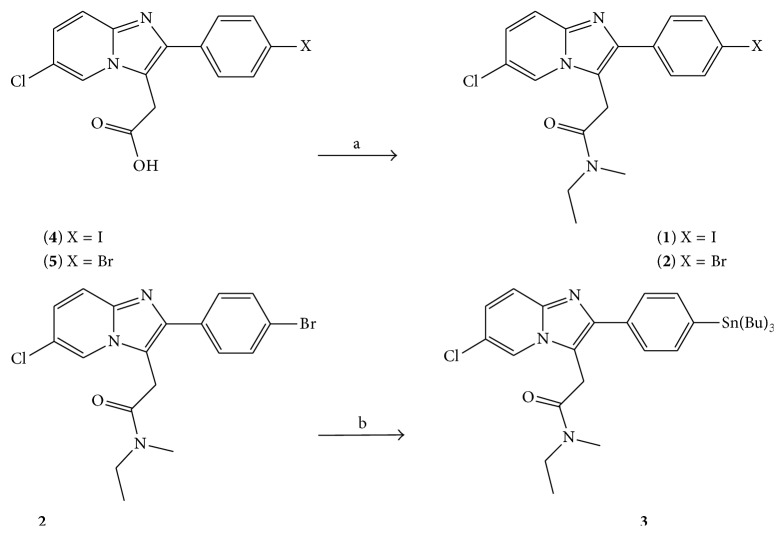
Synthesis of CLINME and stannane precursor (from bromo) for radiolabelling: (a) for** 1**,* N*-methylethylamine, CDI, and THF at 55°C, for** 2** *N*-methylethylamine, EDC, HOBT, in DMF at RT (b) Sn_2_(Bu)_6_, Pd(PPh_3_)_4_ in toluene at reflux.

The synthesis of the radiolabelling precursors and standards followed the scheme in Figure 2.

### 2.2. 2-(6-Chloro-2-(4-iodophenyl)imidazo[1,2-a]pyridin-3-yl)-*N*-ethyl-*N*-methylacetamide (CLINME) (**1**)

Under a nitrogen atmosphere, 1,1′-carbonyldiimidazole (CDI) (1.14 g, 7.0 mmol) was added to a suspension of 2-(6-chloro-2-(4-iodophenyl)imidazo[1,2-a]pyridin-3-yl)acetic acid** 4** [32, 33] (2.32 g, 5.8 mmol) in anhydrous THF (100 mL) and the resultant mixture stirred for 1 h at RT, followed by 1 h at 55°C. After cooling to RT,* N*-methylethylamine (0.38 g, 6.4 mmol) in THF (1 mL) was added. The mixture was stirred for 1 h and the solvent evaporated. The remaining residue was taken up in CH_2_Cl_2_ (100 mL) and washed with 10% Na_2_CO_3_ solution (80 mL) and the aqueous layer extracted with CH_2_Cl_2_ (60 mL × 3). The combined organic layers were dried (Na_2_SO_4_) and evaporated to give the crude product. Recrystallisation from ethyl acetate gave CLINME (**1**).

CLINME** 1** was obtained as a white solid (1.58 g, 60%) (m.p 65–67°C). ^1^H NMR (CDCl_3_): (Rotamers 1 : 1) *δ* 1.06 and 1.13 (t,* J* = 7.2 Hz, 3H, CH_3_), 2.95 (s, 3H, CH_3_), 3.29 and 3.35 (q,* J* = 7.2 Hz, 2H, CH_2_), 4.03 and 4.06 (s, 2H, CH_2_), 7.17 and 7.18 (dd,* J* = 9.5, 1.9 Hz, 1H), 7.38 and 7.39 (d,* J* = 8.4 Hz, 2H), 7.56 (dd,* J* = 9.5, 0.8 Hz, 1H), 7.8 (d,* J*= 8.4 Hz, 2H), 8.21 and 8.23 (*J* = 1.8, 0.8 Hz, 1H). ^13^C NMR (CDCl_3_) d: 12.31, 13.52, 29.66, 30.11, 33.15, 35.05, 43.06, 44.74, 93.94, 93.97, 115.35^*∗*^, 117.75^*∗*^, 120.59, 120.64, 122.48, 122.54, 126.03, 126.08, 130.25^*∗*^, 133.69, 133.76, 137.82^*∗*^, 143.6, 143.61, 143.92, 144.01, 167.03, 167.1. (Note: doublets in carbon spectrum are due to restricted rotation (Rotamers). The numbers with *∗* are the only carbons not appearing as doublets.) MS:* m/z*: 456 (32% [M+H]^+^), 454 (100%, [M+H]^+^). Anal. (C_18_H_17_N_3_OClI) C, H, N.

### 2.3. 2-(2-(4-Bromophenyl)-6-chloroimidazo[1,2-a]pyridin-3-yl)-*N*-ethyl-*N*-methylacetamide (**2**)

Under a nitrogen To DMF (30 mL) under nitrogen atmosphere, were added 2-(2-(4-bromophenyl)-6-chloroimidazo[1,2-a]pyridin-3-yl)acetic acid** 5** [32, 33] (1.0 g, 2.73 mmol),* N*-methylethylamine (0.2 g, 3.38mmol), 4-methylmorpholine (NMM, 1.1 g, 10.9 mmol), 1-hydroxybenzotriazole (HOBT, 0.52 g, 3.38 mmol), and* N*-(3-imethylaminopropyl-N′–ethylcarbodiimide (EDCI, 0.63 g, 4.05 mmol) and then stirred at RT for 24 h. Brine (30 mL) was added and the mixture extracted with ethyl acetate (75 mL × 2). The organic layer was dried (Na_2_SO_4_), filtered, and evaporated to dryness. The solid was triturated with 1 : 5 ethyl acetate/petroleum spirit, filtered, and dried* in vacuo* to obtain (**2**).

The bromo analogue** 2** was obtained as a tan coloured solid (0.61 g, 55%) (m.p 159-160°C). ^1^H NMR (CDCl_3_): (Rotamers 1 : 1) *δ* 1.07 and 1.12 (t,* J* = 7.2 Hz, 3H, CH_3_), 2.95 (s, 3H, CH_3_), 3.28 and 3.44 (q,* J* = 7.2 Hz, 2H, CH_2_), 4.03 and 4.07 (s, 2H, CH_2_), 7.17 and 7.18 (m, 1H, CH), 7.51–7.61 (m, 5H, 4 × ArCH and 1 CH, superimposed), 8.23 (m, 1H, CH). MS (ES+ve)* m/z*: 410 ([M+H]^+^ C_18_H_17_N_3_O^81^Br^37^Cl), 408 ([M+H]^+^ C_18_H_17_N_3_O^81^Br^35^Cl) and ([M+H]^+^ C_18_H_17_N_3_O^79^Br^37^Cl), 406 ([M+H]^+^ C_18_H_17_N_3_O^79^Br^35^Cl). ^13^C NMR (DMSO) d: (12.32, 13.35), (28.41, 28.99), (32.55, 34.50), (42.1, 43.86), 117.42, (117.45, 117.49), (118.82, 118.87), (121.00, 121.06), (125.28, 125.33), (129.66, 129.73), 131.60, (133.43, 133.46), (142.31, 142.34), (142.35, 142.42), 167.18 (note: numbers in parentheses indicate Rotamer pair).

### 2.4. 2-(6-Chloro-2-(4-(tributylstannyl)phenyl)imidazo[1,2-a]pyridin-3-yl)-*N*-ethyl-*N*-methylacetamide (**3**)

To a solution of (**2**) (0.4 g, 0.98 mmol) in toluene (20 mL) were added hexabutylditin (0.86 g, 1.48 mmol) and a catalytic amount of palladium(0) tetrakis(triphenylphosphine) (30 mg, 0.026 mmol). The reaction mixture was heated to reflux for 40 h, cooled, and filtered through celite and the filtrate evaporated to dryness. The resulting brown residue was purified on silica gel eluting firstly with 10% petroleum ether/ethyl acetate followed by 40% petroleum ether/ethyl acetate to give the title compound (**3**).

The stannane labelling precursor** 3** was obtained as a yellow oil (0.3 g, 50%). ^1^H NMR (CDCl_3_): *δ* (Rotamers ~ 55 : 45) 0.88−1.60 (m, 30H, Bu-H and CH_3_, superimposed); 2.95 (s, 1.65H, N-CH_3_), 2.96 (s, 1.35H, NCH_3_); 3.26 (q,* J* = 7.2 Hz, 1.1H, N-CH_2_), 3.46 (q,* J* = 7.2 Hz, 0.9H, N-CH_2_); 4.10 (s, 0.9H, CH_2_), 4.13 (s, 1.1H, CH_2_), 7.30 (m, 1H, CH), 7.61 (s, 4H, ArCH × 4), 7.83 (m, 1H, CH), 8.32 (s, 0.45H, CH), 8.36 (s, 0.55H, CH). MS (ES+ve)* m/z*: major peak 618 ([M+H]^+^ C_30_H_44_N_3_O^35^Cl^120^Sn).

### 2.5. Radiochemistry Analytical Methods

Semipreparative radio-High Pressure Liquid Chromatography (HPLC) was performed on a Berthold LB501 system equipped with a Waters 510 pump, a Spectrophysics-Linear UV detector, set at 254 nm, and an online NaI-Berthold radioactivity *γ*-detector, using a Phenomenex Bondclone C18 column (10 *μ*m, 300 × 7.8 mm). The analytical HPLC system was equipped with a Varian 9002 pump with a Spectrophysics-Linear UV detector set at 230 nm and 254 nm and an Acemate *γ*-detector using a Phenomenex Synergi hydro C18 column (4 *μ*m 250 × 4.6 mm).

### 2.6. Radiolabelling and Quality Control of [^123/125^I]-CLINME

The tributyltin precursor (0.25 mg, 0.40 *μ*mol) in acetic acid (100 *μ*L) was treated with a solution of Na^123^I (ANSTO Health) or Na^125^I (GE-Healthcare) (0.5–1.1 GBq) followed by peracetic acid (32%, 50 *μ*L). After 5 min the solution was quenched (Na_2_S_2_O_5_) (4.9 mg, 30 *μ*mol, in 100 *μ*L H_2_O), neutralised with NaHCO_3_ (5.5 mg, 65 *μ*mol, in 100 *μ*L H_2_O), and diluted with mobile phase (300 *μ*L). The resulting mixture was purified by semipreparative radio-HPLC, eluting with a mobile phase consisting of acetonitrile : ammonium acetate (0.01 M) (50 : 50) at a flow rate of 4 mL/min. For radiochemical purity and stability measurements, the radioiodinated solution was ascertained on an analytical HPLC system, eluting with acetonitrile : 0.1 M ammonium acetate (60 : 40) at a flow rate of 1 mL/min.

### 2.7. Lipophilicity

The log *P* values were estimated by comparing HPLC retention times of test compounds with retention times of standards having known log *P* values as described [34].

### 2.8. Biological Evaluation

All* in vivo* experimental procedures were carried out in compliance with Australian laws governing animal experimentation.

### 2.9. *In Vitro* Binding Assays

Inhibition constant (IC_50_) of CLINME for the TPSO and for the central benzodiazepine receptor (CBR) was determined as described previously [35]. The IC_50_ values were converted to the inhibition constant, Ki, using Cheng-Prusoff equation.

### 2.10. *In Vivo* Biodistribution Studies

No carrier added [^123^I]-CLINME (0.7 MBq, 10 pmoles) dissolved in 0.9% saline (0.1 mL) was administered to 8–10-week-old male Sprague-Dawley (SD) rats (*n* = 4–6) via tail vein injection in a volume of 0.1 mL. At 5, 15, and 30 min and 1, 3, and 6 h after injection (p.i.) of the [^123^I]-CLINME, the rats were sacrificed by CO_2_ administration followed by cervical fracture. Peripheral organs (including liver, spleen, thyroid, pancreas, lung, heart, kidney, adrenals, and blood), olfactory bulbs, and the remainder of the brain were removed. Samples of organs and tissues were weighed and the radioactivity was measured using an automated *γ*-counter. The percent of injected dose (%ID) was calculated by comparison to a diluted standard solution derived from the initial injected sample. Radioactivity concentrations were expressed as percent of injected dose per gram of wet tissue (% ID/g).

### 2.11. Pharmacological Competition Studies

The saturation effect of [^123^I]-CLINME uptake was studied in competition experiments by injecting CLINME (1 mg/Kg), 5 min prior to [^123^I]-CLINME (0.7 MBq, 10 pmoles in 0.1 mL of 0.9% saline), via the tail vein, to 8–10-week-old male SD rats.

The* in vivo* specificity of [^123^I]-CLINME uptake in rats was investigated in competition experiments using flumazenil (CBR selective drug), Ro 5-4864, and PK11195 (TPSO selective drugs) (1 mg/Kg). The drugs were dissolved in dimethyl sulfoxide/saline (1 : 5) and injected intravenously 5 min prior to [^123^I]-CLINME. Groups of rats (*n* = 3-4) were sacrificed by CO_2_ administration followed by cervical fracture 30 min after administration of [^123^I]-CLINME and peripheral organs (liver, spleen, lung, heart, kidney, and adrenals), blood, olfactory bulbs, and the remainder of the brain were removed. The radioactivity concentrations were calculated as described previously. Results were analysed by one-way analysis of variance (ANOVA) followed by Tukey's post hoc test for comparing the tissue radioactivity concentrations in the treated animals (*n* = 3-4) and in the controls (*n* = 6). The criterion for significance was *P* < 0.05.

### 2.12. Metabolite Studies

Groups of male SD rats were injected with [^123^I]-CLINME (7.4 MBq in 0.1 mL of 0.9% saline) and sacrificed at 15, 60, and 180 min p.i. Adrenals, kidney, heart, and brain were quickly excised.

Tissue fractions (200 mg) were treated with acetonitrile (2 mL) and homogenised, and the mixture was centrifuged at 2,000 rpm. The supernatant was removed and the pellet was treated for a second time, as above and the combined supernatants concentrated to 50 *μ*L. Plasma (0.2 mL) separated from blood was extracted with acetonitrile and concentrated. The concentrated extracts were applied onto silica gel C18 plates with a preconcentration zone (Merck, Darmstadt, Germany) and developed with ethyl acetate. The radioactivity distribution was measured using a static radiochromatogram analyser (Berthold Co.).

### 2.13. Unilateral Excitotoxic Lesion

Lesioning was performed by stereotaxically injecting 7.5 nmol *α*-amino-3-hydroxy-5-methylisoxazole-4-propionic acid (AMPA) unilaterally into the striatum. Male SD rats (317 ± 56 g) were anaesthetised with inhaled isoflurane in O_2_ (5% induction, ~2% maintenance thereafter) and positioned in a stereotaxic frame (Stoelting Co., USA). A 1.5 cm incision was made in the scalp to reveal the skull surface and a burr hole was drilled over the injection site. Animals were injected with AMPA (15 mM) in a volume of 0.5 *μ*L of 0.9% saline using a Hamilton syringe in a microinfusion pump (KD Scientific Co., USA) at a rate of 0.5 *μ*L/min (*n* = 5, coordinates: AP = +0.7 mm and* L* = ±2.7 mm (left *n* = 2, right *n* = 3), relative to bregma; DV = −5.5 mm from dura) [36]. Following the injection, the needle was left* in situ* for a further 5 min to allow the fluid to disperse. After the needle was withdrawn, the incision was sutured and cleansed. Animals were given a week to recover prior to imaging. Analgesia was provided by subcutaneous administration of 0.05 mg/Kg buprenorphine.

### 2.14. SPECT Imaging

Two hours prior to [^123^I]-CLINME administration, thyroid blocking was performed with an intraperitoneal injection of 2 mg/Kg potassium iodide. [^123^I]-CLINME (74–148 MBq in 0.1 mL of 0.9% saline, S.A. > 80 GBq/*μ*mol) was injected via the tail vein. One hour p.i., the animals were imaged on a dual-head *μ*-SPECT system (GammaMedica, USA) equipped with medium-energy pinhole collimators (focal length 90.0 mm, aperture diameter 1.5 mm), under isoflurane anaesthesia (1-2% in O_2_). Two acquisitions (each of 64 projections, 60 seconds per projection, 4.5 cm ROR) were performed sequentially. The energy window was 20%, centred over 159 keV. Following the SPECT, an X-ray Computed Tomography (CT) scan was performed (voltage 70 kVp).

SPECT volumes were reconstructed with an Ordered Subset Expectation Maximisation iterative reconstruction algorithm using 3 iterations and 8 subsets. Due to the expected low-level of activity in the brain, as a function of the known low-level of TSPO in the CNS, a 20x scaling was applied to the SPECT images post hoc to maximise the dynamic range. The SPECT and CT volumes were coregistered, displayed, and analysed using Anatomist visualisation software [37]. An ipsilateral volume of interest (VOI, 24.4 mm^3^) was created in the dorsal striatum, using the CT-visualised burr hole as a landmark, and a corresponding VOI placed on the contralateral side as the control region. At each of the time points p.i., the ratio of the mean activity in the lesion VOI compared to the contralateral control VOI was determined. The data were compared using the nonparametric Mann-Whitney* U* test with a *P* value < 0.05 considered significant.

Following imaging, the animals were euthanised by CO_2_ administration and the brains removed and frozen with liquid nitrogen. Tissue was stored at −80°C, cut (20 *μ*m), and mounted onto poly-L-lysine coated slides and then stored at −80°C until use for autoradiography and immunohistochemistry.

### 2.15. Autoradiography

Autoradiography with [^125^I]-CLINME was performed on brain tissue. Nonspecific binding was assessed by coincubating the slides with 20 *μ*M of either PK11195 or CLINME. The specificity of [^123^I]-CLINME for the TSPO was assessed by coincubating slides with flumazenil (20 *μ*M). Sections were incubated at RT for 1 h in 100 mM Phosphate-Buffered Saline (PBS, pH 7.4, and 0.9% NaCl) containing 16 nM [^125^I]-CLINME, either alone or with 20 *μ*M of one of the drugs above. They were then rinsed twice in ice-cold buffer and once in distilled H_2_O, dried overnight, exposed for 3 h to film (Hyperfilm, Amersham Biosciences, UK; Kodak Biomax MR, Sigma-Aldrich, USA), then developed, fixed, and dried.

Images were digitised and analysed using MCID image analysis software. To allow comparison with the SPECT images, a 2D region of interest (ROI) was placed over the dorsal striatum, with corresponding ROI placed on the contralateral side as a control region, the square ROIs having the same area as one facet of the cubic VOIs used to analyse the SPECT images. The mean relative optical density (ROD) was then determined in each region. Mean ROD in the ipsilateral versus the control ROI within each of the 4 incubation conditions ([^125^I]-CLINME alone or coincubated with one of the 3 blocking drugs) was compared using the nonparametric Mann-Whitney* U* test. Differences between the coincubated slides and those incubated with [^125^I]-CLINME alone were compared separately for lesion and control ROIs using the nonparametric Kruskal-Wallis test and post hoc Mann-Whitney* U* tests. A *P* value < 0.05 was considered significant.

### 2.16. Immunohistochemistry and Immunofluorescence

Immunohistochemistry was performed in the same manner as described by Boutin et al. [31]. Sequential sections of tissue, adjacent to those used for autoradiography, were stained either for glial fibrillary acidic protein (GFAP) and CD11b antigen (Ox42) or for GFAP and neuron-specific nuclear protein (NeuN). Sections were fixed in 10% formalin (Sigma-Aldrich, USA) for 1 h, washed in 6 × 5 min PBS, and then incubated for 20 min in diluent (PBS with 5% normal horse serum and 0.1% Triton X-100). Sections were then incubated overnight at 4°C with primary antibodies in diluent (rabbit anti-cow GFAP, 1 : 1,000, DakoCytomation; mouse anti-rat CD11b, 1 : 1,000, Serotec; mouse anti-mouse NeuN, 1 : 1,000, Chemicon) and then washed in 3 × 5 min PBS. Incubation in secondary antibodies was for 1 h at RT (Alexa Fluor 594 nm goat anti-mouse IgG, 1 : 500; Alexa Fluor 488 nm goat anti-rabbit IgG, 1 : 500 (Molecular Probes, Invitrogen)) and then sections were washed again in 3 × 5 min PBS. Sections were mounted with an antiadherent solution (Citifluor, UK). Sections incubated without primary antibodies served as negative controls.

Immunofluorescence images were obtained with an Olympus BX-41 microscope equipped with a digital camera (QImaging, Canada) at 10x magnification, acquired with Image-Pro software. Image fusion was performed with MCID image analysis software.

## 3. Results

### 3.1. Lipophilicity

The lipophilicity of CLINME was ascertained to understand the* in vivo* uptake of [^123^I]-CLINME. The CLINME log* P*_7.5_ value was 3.27 in comparison with the log* P*_7.5_ of PK11195 that was 3.35.

### 3.2. *In Vitro* Binding Assays

Data from the binding assays on membranes from kidney and brain cortex gave *K*_*i*_ values for the TSPO of 1.6 ± 0.2 nM ([^3^H]-PK11195) and 4.5 ± 0.6 nM ([^3^H]-Ro 5-4864). The inhibition constant for the CBR was 183 ± 20 nM.

### 3.3. Radiolabelling

[^123/125^I]-CLINME was prepared by electrophilic substitution of the tributyltin precursor with ^123/125^I-NaI in acetic acid using peracetic acid as the oxidant followed by HPLC purification. [^123/125^I]-CLINME was eluted with a retention time *k*′ = 14 min in a radiochemical yield of 74 ± 5% (*n* = 8) and 86 ± 8% (*n* = 4) for [^123^I]- and [^125^I]-CLINME, respectively. Radiochemical purity as assessed by analytical HPLC was >99% and the specific activity (SA) for [^123^I]-CLINME was >185 GBq/*μ*mol based on the limit of detection of the HPLC system. The SA for [^125^I]-CLINME was measured as 80 GBq/*μ*mol close to the theoretical specific activity of the ^125^I-iodine. [^123^I]-CLINME was reconstituted in saline for* in vivo* pharmacological studies. The radiochemical purity of [^123^]-CLINME in saline at room temperature, 3 h after synthesis, was >97% whereas [^125^I]-CLINME was stable (>97%) for up to 6 weeks in ethanol when stored at −80°C.

For* in vitro* assays, CLINME was added to the [^125^I]-CLINME to achieve a specific activity of 3.7 GBq/*μ*mol and reconstituted in ethanol.

### 3.4. Biodistribution

Table 1 summarises the tissue time course distribution of [^123^I]-CLINME in rats. In the peripheral organs, a plateau was observed in the adrenals after 30 min to 6 h p.i. (≈7.5% ID/g). In liver, spleen, kidney, heart, and lung the activity peaked (0.7, 2.7, 1.8, 3.5, and 7.9% ID/g) at 5 or 15 min p.i. and decreased over time to less than 0.7% ID/g at 6 h. Very low concentrations of [^123^I]-CLINME were found in blood (<0.18% ID/g) throughout the time of measurement. In the olfactory bulbs (0.38–0.17%), a significantly higher concentration of the radioactivity was found compared to the remainder of the brain (0.2–0.02%), with ratios increasing from 2 at 5 min to 8 at 6 h. The uptake in the thyroid increases from 5 min to 1 h (4–7%) and then a decrease was observed at 3 h to 4% which remained constant till 6 h.

### 3.5. Pharmacological Competition Studies

The saturating effect of CLINME (1 mg/Kg) was observed with high significant decreases in the heart, kidney, lungs, and olfactory bulbs by 86, 71, 76, and 38% (*P* < 0.01), respectively. A nonsignificant reduction of uptake was also observed in the adrenals. A significant increase (*P* < 0.01) of [^123^I]-CLINME uptake was observed in the blood (45%).

**Figure 3:**
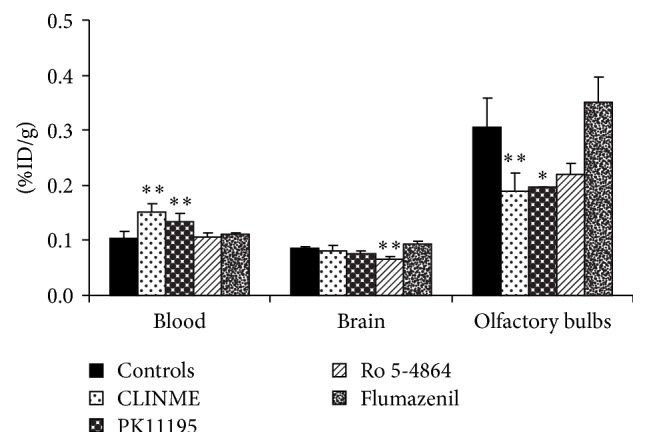
Effect of competing drugs (CLINME, PK11195, Ro 5-4864, and flumazenil, 1 mg/Kg) on [^123^I]-CLINME uptake in CNS and blood of normal rat. Results are average of 3–6 rats ± SD, 30 min p.i. of [^123^I]-CLINME. Unit is percent injected dose per gram (%ID/g) (^*∗∗*^ *P* < 0.01, ^*∗*^ *P* < 0.005).

**Figure 4:**
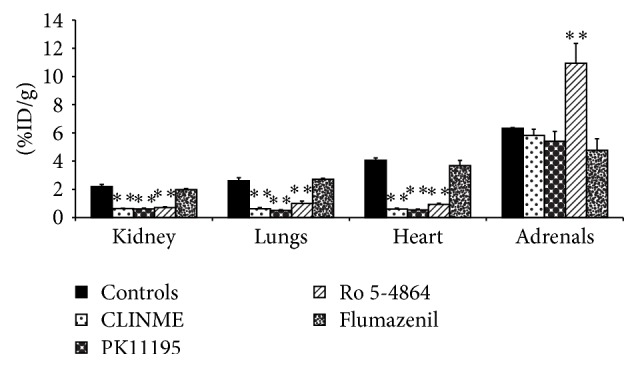
Effect of competing drugs (CLINME, PK11195, Ro 5-4864, and flumazenil, 1 mg/Kg) on [^123^I]-CLINME uptake in peripheral organs of normal rat. Results are average of 3–6 rats ± SD, 30 min p.i. of [^123^I]-CLINME. Unit is percent injected dose per gram (%ID/g) (^*∗∗*^ *P* < 0.01).

Drug competition experiments were carried out to test the ability of TSPO and CBR drugs to inhibit [^123^I]-CLINME uptake in the brain and in peripheral organs (Figures 3 and 4). PK11195 was the most potent in inhibiting the uptake of [^123^I]-CLINME in the heart, lungs, and kidney by 87, 81, and 72% (*P* < 0.01), respectively, compared to control animals. A significant decrease (*P* < 0.05) of 36% was also observed in the olfactory bulbs. A nonsignificant reduction was observed in the adrenals. With this drug, the uptake of [^123^I]-CLINME in blood was significantly increased by 29% compared to controls (*P* < 0.01). Ro 5-4864 also significantly reduced the uptake of activity in the heart, kidney, lungs, and the remainder of the brain by 77, 68, 62, and 23% (*P* < 0.01), respectively. A nonsignificant reduction was observed in the olfactory bulbs. The reduction of uptake of [^123^I]-CLINME in the blood was not significant, while the concentration in the adrenals was significantly increased by 73% compared to controls (*P* < 0.01). With flumazenil nonsignificant differences of [^123^I]-CLINME uptake in peripheral organs and brain were observed.

### 3.6. Metabolite

The time course of unchanged [^123^I]-CLINME was studied in adrenals, kidney, brain, heart, and olfactory bulbs as well as in plasma. With the homogenisation technique used in this experiment, more than 85% of the activity from tissues and plasma was extracted into acetonitrile and analysed by radio thin layer chromatography. In the adrenals, kidney, and heart, more than 95% of the extractable radioactivity was shown to be unchanged [^123^I]-CLINME, which remained constant over the three hours studied. Brain tissue, even the low activity present, represents >95% of unchanged radiotracer. In plasma, metabolite analysis showed that 15, 5, and 2% of the extracted activity were unchanged tracer, 15 min and 1 and 3 h p.i., respectively.

### 3.7. SPECT Imaging

**Figure 5:**
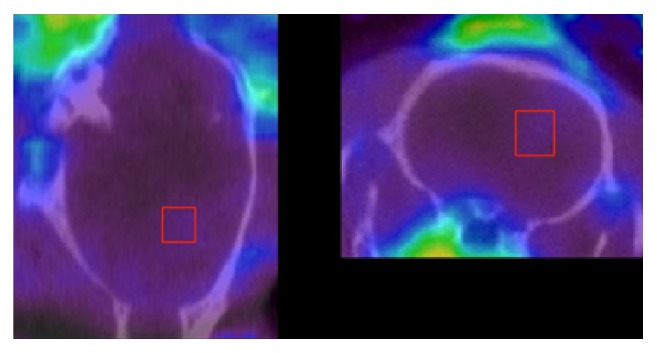
SPECT images of [^123^I]-CLINME uptake in the unilateral excitotoxic lesion rat model. The red square represents the volume of interest (VOI) used to sample the activity. A corresponding region was drawn on the contralateral side as a control (region not shown).

SPECT imaging with [^123^I]-CLINME showed a significantly higher signal in the area of the lesion than in the corresponding control striatum at both time points p.i. (Figure 5). The mean ratio ± SD (ipsilateral : contralateral) found with SPECT at 60 min p.i. was 2.18 ± 0.89 and 1.86 ± 0.51 at 120 min p.i.

### 3.8. Autoradiography

**Figure 6:**
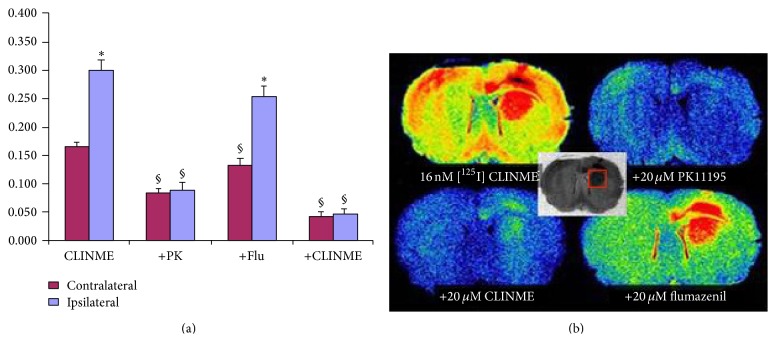
Measures of relative optical density (ROD) following* in vitro* autoradiography with 16 nM [^125^I]-CLINME and effect of 20 *μ*M of PK11195, CLINME, or flumazenil (a). Representative autoradiograms showing a region of very high activity in the unilateral dorsal striatum (with extension dorsally into the cortex). Specific binding in the lining of the lateral ventricles can also be observed (b). ^*∗*^ *P* < 0.05 ipsilateral versus contralateral; ^§^ *P* < 0.05 versus [^125^I]-CLINME alone.

In tissue harvested immediately following SPECT imaging, autoradiography with 16 nM [^125^I]-CLINME also showed significantly increased radioligand binding in the lesioned striatum compared to the contralateral one (Figure 6). This differential in [^125^I]-CLINME binding was eliminated by coincubation of [^125^I]-CLINME with 20 *μ*M of either CLINME or PK11195. Coincubation with 20 *μ*M flumazenil on the other hand had no effect on the differential binding of [^125^I]-CLINME. Analysis of the ipsilateral data across the different incubation conditions shows that the two TSPO drugs greatly and significantly decreased the measured ROD in these ROIs, whilst coincubation with flumazenil produced a small, nonsignificant reduction. In the contralateral striatum, all 3 coincubation conditions resulted in significantly decreased ROD compared to incubation with 16 nM [^125^I]-CLINME alone, although these decreases were of a smaller magnitude than those observed ipsilaterally.

### 3.9. Immunohistochemistry

**Figure 7:**
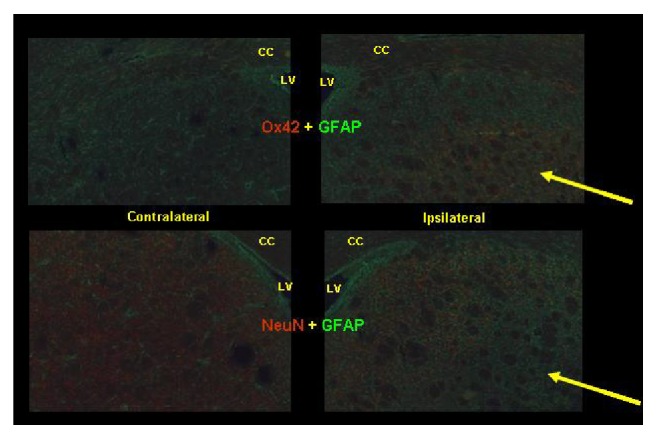
Immunohistochemistry for glial fibrillary acidic protein (GFAP, green) and anti-CD11b antibody (Ox42) or neuron-specific nuclear protein (NeuN, both red). The arrows indicate a region identified as the excitotoxic lesion, with increased Ox42 staining and decreased NeuN staining indicating microglial activation and neurodegeneration, respectively. LV = lateral ventricle, CC = corpus callosum.

Immunohistochemistry using primary antibodies targeting astrocytes (GFAP), microglia (Ox42), and neurons (NeuN) demonstrated the presence of the excitotoxic AMPA lesion with a pattern of staining consistent with that reported in the earlier ([^11^C]-CLINME) study with this same model [31]. Tissue harvested immediately following SPECT imaging stained strongly throughout the imaged contralateral striatum for NeuN. In the medial part of the ipsilateral striatum NeuN staining was still present; however it was almost completely absent centrally. GFAP staining in these animals was present in both hemispheres, though it was moderately increased on the lesioned side, whilst Ox42 staining was barely perceptible in most regions, except in the region where NeuN immunoreactivity was virtually absent (Figure 7).

## 4. Discussion

In the present study we have prepared, radiolabelled, and evaluated* in vivo* the novel imidazopyridine derivative, CLINME, to determine its selectivity for the TPSO.* In vitro* studies found that CLINME displayed a 110-fold higher selectivity for the TPSO over the CBR compared to a 20-fold difference to the related compounds, alpidem [38], and 200-fold for CLINDE [35]. Furthermore the presence of the iodine atom and the* N*-methyl group on the imidazopyridine structure presented the opportunity for radiolabelling with either radioiodine or carbon-11 without loss in affinity or selectivity for the TPSO, making this analogue useful for both PET and SPECT imaging.

*In vivo* the distribution of radioactivity after injection of [^123^I]-CLINME follows the distribution of the TPSO reported in the literature with high uptake in endocrine tissues, such as adrenal glands [39], in peripheral organs such as kidney and heart [17, 40] and low uptake in the brain [41, 42]. In the brain, the highest density of TPSO reported is in the olfactory bulbs [4]; this correlates with the results obtained with [^123^I]-CLINDE [43] and other radiolabelled radiotracers belonging to the* N*-benzyl-*N*-(2-phenoxyaryl)-acetamide series (DAA tracers) [44, 45]. The ratio of uptake of [^123^I]-CLINME in the olfactory bulbs versus the rest of brain increased from 2- to 8-fold between 5 min and 6 h; this ratio is more than twice that reported for [^18^F]FE-DAA1106 of 3.5 and comparable with [^123^I]-CLINDE (6 at 8 h). The uptake in the olfactory bulbs is also always much higher than that of the blood (ratio 6 : 1) indicating that the uptake in the brain is not due to blood flow but to binding to TPSO sites. In addition, metabolite analysis confirmed that the uptake in the brain was due to intact [^123^I]-CLINME. These results indicated that [^123^I]-CLINME crosses the brain-blood barrier (BBB), a prerequisite for brain imaging.

The pharmacological competition studies confirm that the uptake in the olfactory bulbs and peripheral organs could be blocked by PK11195, Ro 5-4854, and CLINME indicating that the uptake was specific to TPSO binding sites. However, the uptake in the adrenals could not be blocked by CLINME or PK11195 (1 mg/Kg) whereas an increase in uptake was found with Ro 5-4864. These observations could be attributed to the much higher density of TPSO in the adrenals, the amount of competing drugs used (since a dose response curve was not determined, it is possible that a higher dose than 1 mg/Kg might result in greater inhibition of uptake), or the decreased availability of TSPO binding sites for [^123^I]-CLINME in the peripheral organs that are occupied by the competing drugs. This observation was also found with carbon-11 and fluorine-18-labelled analogues belonging to the dimethylpyrazolo[1,5-a]pyrimidin-3-yl)acetamide (DPA) series [46] whereas other studies using labelled quinoline carboxamides suggested that the uptake in the adrenals was nonspecific [47]. Gildersleeve et al. reported a dose of 5 mg/Kg of PK11195 to block the uptake of [^123^I]-PK11195 by 85% in the adrenals of rats [17] and Mattner et al. reported a dose of 10 mg/Kg to block the uptake of [^123^I]-CLINDE by 51% [43]. As expected flumazenil was unable to inhibit the uptake of [^123^I]-CLINME in peripheral organs or CNS.

The novel TSPO radioligand, [^123^I]-CLINME, was also investigated in a rat model of neuroinflammation following excitotoxic lesioning of the striatum. In previous PET studies [^11^C]-CLINME was shown to be superior to [^11^C]-PK11195 [30, 31]; similarly the [^123^I]-CLINME SPECT analogue showed comparable results in the same animal model, which was confirmed with immunohistochemistry (Figure 7). The ratio of [^123^I]-CLINME observed in the lesion versus contralateral striatum (2.18 ± 0.89) was consistent with that found in the same animal model with [^11^C]-CLINME PET (2.14 ± 0.09) and considerably higher than [^11^C]-PK11195 (1.62 ± 0.05) [31].

The high relative accumulation of [^123^I]-CLINME in the lesion versus contralateral striatum visualised with SPECT was observed with* in vitro* autoradiography of [^125^I]-CLINME alone (Figure 6). The differential accumulation was eliminated by coincubation with TSPO ligands, but not by the flumazenil. Confirming this, analysis of the ipsilateral (lesion) ROIs showed sections coincubated with the TSPO ligands, but not flumazenil, to have significantly lower RODs than those in sections incubated with [^125^I]-CLINME alone.

The large increase in TPSO expression associated with the lesion, as demonstrated by [^123/125^I]-CLINME, was colocalised with the large increase in CD11b expression, moderate increase in GFAP, and large decrease in NeuN visualised with immunohistochemistry (Figure 7). CD11b is a *β*-2 integrin, present in resting microglia, which is known to have greatly increased expression following microglial activation [48]. In turn, microglial activation is the primary source of increased TSPO density following the neurological insult, as opposed to reactive astrocytes (which were demonstrated here with GFAP to be moderately increased) [5, 7]. The loss of NeuN indicates neuronal loss subsequent to the excitotoxic AMPA infusion. Taken together, it is strongly suggested that the putative increase in TSPO expression demonstrated with SPECT and autoradiography was due to activated microglia induced by AMPA infusion, though it cannot be ruled out that some proportion of the TSPO number in the lesion site was due to invasion of peripheral TSPO expressing immune cells across a compromised BBB [49] and CD11b [48]. The possibility of BBB being compromised was addressed in the PET study of [^11^C]-CLINME in this animal model which showed, via assessment of Evan's Blue extravasation in a parallel group of animals, that the BBB was intact 7 days after AMPA administration to the striatum [31].

The present study has demonstrated that SPECT with [^123^I]-CLINME is able to provide visualisation of a unilateral excitotoxic lesion with a sensitivity similar to that reported for PET with [^11^C]-CLINME. The uptake ratio that both radioligands provided between the lesion and the contralateral control tissue, as imaged with their respective technologies, was superior to that found with [^11^C]-PK11195 PET in this model [31]. These results indicate that [^123^I]-CLINME may be a very useful radiotracer for the SPECT imaging of microglial activation associated with various neuropathologies. The rapidity of microglia activation following a variety of neurological insults and the sensitivity of this process to even subtle changes in addition to its implication in many pathogenic processes prompts the* in vivo* imaging of microglia activation with TSPO.

## 5. Conclusion

[^123/125^I]-CLINME can be conveniently prepared via an iododestannylation reaction in high specific activity suitable for imaging microglial activation.* In vitro* studies confirmed that CLINME exhibits high affinity and selectivity for the TSPO.* In vivo*, [^123^I]-CLINME indicated a biodistribution pattern consistent with known TPSO distribution with high selectivity for the TSPO as demonstrated by competition studies with PK11195 and Ro 5-4864. Furthermore, imaging studies showed that [^123^I]-CLINME, in an AMPA induced excitotoxic model, is a promising new radiotracer for the quantification and visualization of TPSO expression in activated microglia with SPECT. Finally, CLINME is a versatile radiotracer that can be prepared for PET (^11^C/^124^I) or SPECT (^123^I) imaging as well as for biochemical and pharmacological studies using the ^125^I-analogue.

## Figures and Tables

**Table 1:** Biodistribution of [^123^I]-CLINME in SD male rats.

%ID/g	5 min	15 min	30 min	1 hour	3 hours	6 hours
Liver	0.70 ± 0.10	0.68 ± 0.07	0.51 ± 0.07	0.42 ± 0.07	0.23 ± 0.01	0.21 ± 0.05
Spleen	2.00 ± 0.55	2.65 ± 0.40	2.21 ± 0.15	1.76 ± 0.12	1.02 ± 0.14	0.67 ± 0.09
Kidney	1.82 ± 0.20	1.75 ± 0.07	1.78 ± 0.18	1.16 ± 0.18	0.70 ± 0.05	0.51 ± 0.09
Lungs	7.88 ± 0.62	3.53 ± 0.44	2.45 ± 0.30	1.68 ± 0.10	0.95 ± 0.12	0.68 ± 0.08
Heart	3.28 ± 0.30	3.53 ± 0.23	3.32 ± 0.31	2.601 ± 0.13	1.28 ± 0.13	0.72 ± 0.14
Blood	0.18 ± 0.02	0.09 ± 0.01	0.08 ± 0.01	0.06 ± 0.00	0.04 ± 0.00	0.03 ± 0.00
Brain	0.20 ± 0.02	0.10 ± 0.01	0.08 ± 0.00	0.06 ± 0.01	0.03 ± 0.00	0.02 ± 0.00
Olfactory bulbs	0.38 ± 0.03	0.28 ± 0.04	0.34 ± 0.01	0.26 ± 0.05	0.25 ± 0.01	0.17 ± 0.05
Thyroid	4.62 ± 2.17	5.62 ± 1.24	7.37 ± 2.14	6.46 ± 2.26	3.41 ± 1.10	4.11 ± 1.15
Adrenals	2.89 ± 0.48	4.71 ± 0.26	7.55 ± 1.38	6.77 ± 0.54	6.65 ± 0.56	8.34 ± 0.71

Results are mean ± SD percent of injected dose per gram (%ID/g), *n* = 3-4 rats.
